# Epigenetic and Posttranslational Modifications in Light Signal Transduction and the Circadian Clock in *Neurospora crassa*

**DOI:** 10.3390/ijms160715347

**Published:** 2015-07-07

**Authors:** Marco Proietto, Michele Maria Bianchi, Paola Ballario, Andrea Brenna

**Affiliations:** 1 Department of Biology and Biotechnologies “Charles Darwin”, Sapienza-University of Rome, Piazzale Aldo Moro 5, Rome 00185, Italy; E-Mails: marco.proietto@uniroma1.it (M.P.); michele.bianchi@uniroma1.it (M.M.B.); paola.ballario@uniroma1.it (P.B.); 2 Pasteur Institute, Cenci Bolognetti Foundation and Department of Biology and Biotechnology “Charles Darwin”, Sapienza-University of Rome, Piazzale Aldo Moro 5, Rome 00185, Italy; 3 Department of Biology, Division of Biochemistry, University of Fribourg, Chemin du Musée 5, Fribourg 1700, Switzerland

**Keywords:** phosphorylation, acetylation, methylation, chromatin remodeling, light signal transduction, circadian rhythms, *Neurospora crassa*

## Abstract

Blue light, a key abiotic signal, regulates a wide variety of physiological processes in many organisms. One of these phenomena is the circadian rhythm presents in organisms sensitive to the phase-setting effects of blue light and under control of the daily alternation of light and dark. Circadian clocks consist of autoregulatory alternating negative and positive feedback loops intimately connected with the cellular metabolism and biochemical processes. *Neurospora crassa* provides an excellent model for studying the molecular mechanisms involved in these phenomena. The White Collar Complex (WCC), a blue-light receptor and transcription factor of the circadian oscillator, and Frequency (FRQ), the circadian clock pacemaker, are at the core of the *Neurospora* circadian system. The eukaryotic circadian clock relies on transcriptional/translational feedback loops: some proteins rhythmically repress their own synthesis by inhibiting the activity of their transcriptional factors, generating self-sustained oscillations over a period of about 24 h. One of the basic mechanisms that perpetuate self-sustained oscillations is post translation modification (PTM). The acronym PTM generically indicates the addition of acetyl, methyl, sumoyl, or phosphoric groups to various types of proteins. The protein can be regulatory or enzymatic or a component of the chromatin. PTMs influence protein stability, interaction, localization, activity, and chromatin packaging. Chromatin modification and PTMs have been implicated in regulating circadian clock function in *Neurospora*. Research into the epigenetic control of transcription factors such as WCC has yielded new insights into the temporal modulation of light-dependent gene transcription. Here we report on epigenetic and protein PTMs in the regulation of the *Neurospora crassa* circadian clock. We also present a model that illustrates the molecular mechanisms at the basis of the blue light control of the circadian clock.

## 1. The *Neurospora crassa* Light Responses

### 1.1. Light Signal Transduction

Exposure to environmental light stimulates physiological responses in all living organisms, from prokaryotes to eukaryotes [1]. Studies in plants have identified photoreceptors that respond to two types of light signals: blue light and red light [2,3,4]. Although plants were the first organisms in which molecular mechanisms of light signal transduction were characterized, the Ascomyceta filamentous fungus *Neurospora crassa* represents the most used model system. *N. crassa* is sensitive only to blue light, although genes coding for other putative photoreceptors have been identified [5,6]. Nevertheless, it seems that the deletion of these genes does not cause aberration in response to blue light stimulation [7]. Blue light exposure induces many different physiological responses in *N. crassa*, including entrainment, biosynthesis of photo protective pigments, induction of asexual conidiospores formation, phototropism of peritecial beaks, development of sexual structures, and the direction of ascospore dispersal [8,9,10,11,12,13]. Several genes coding for proteins activated in response to light stimulation have been identified [14,15]. Genome-wide analysis shows that approximately 5.6% of the protein-coding genes in *N. crassa* are responsive to light [16]. Light-sensitive genes are grouped in two large classes: early light-responders (ELR), with average peaks in mRNA expression at approximately 15 to 30 min, and late light-responders (LLR) in which mRNA expression peaks between 60 and 90 min [17].

### 1.2. White Collar Complex (WCC) Activation

**Figure 1:**
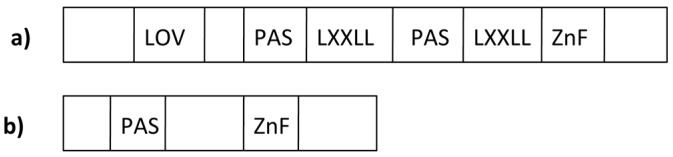
Domains composing White Collar 1 (**a**) and White Collar 2 (**b**). Legend: LOV domain: Light-Oxygen-Voltage sensitive domain; LXXLL: WC-1/NGF1 interaction domains; PAS domain: Per Arnt Sim domain dimerization domain; Zn finger: Zinc finger, DNA binding domain.

The light signaling system is regulated by the White Collar complex (WCC). The WCC consists of a heterodimer formed by the product of the *white collar-1* (*wc-1*) and *white collar-2* (*wc-2*) genes [18,19]. WC-1 and WC-2 contain Per-ARNT-Sim (PAS) domains, which serve as versatile sensor and interaction modules in signal transduction for protein-protein interaction [20]. The WC-1 first PAS is a modified domain known as the light-oxygen-voltage domain (LOV), which is a PAS domain variant initially found in phototropins, a family of blue-light sensitive plant photoreceptors (Figure 1a) [21]. This domain is able to bind chromophores such as Flavin Adenine Dinucleotide (FAD) makingWC-1 a “photoreceptor”: when a chromophore captures photons, it generates a photo-adduct with the LOV domain, which changes WC-1 conformation and promotes signal transmission [22]. Like its molecular partner WC-1, WC-2 shares the same functional domains except the LOV domain (Figure 1b).

WC-1 heterodimerizes with WC-2 to form the WCC, which then translocates into the nucleus where it mediates conformational changes when a light signal is perceived and activates the transcription of hundreds of target genes [23]. Both WC-1 and WC-2 proteins can bind promoter sequences called Light Responsive Elements (LREs) by their zinc-finger motifs. The LREs promoter sequences contain GATC/G consensus sequences. These regions were recognized by *in vitro* binding assays using recombinant WC-1 and WC-2 zinc-finger domains or activated WCC [24]. WCC is able to homodimerize via the activated LOV domain and binds preferentially to tandem GATC motifs in the presence or absence of light [24,25]. Nevertheless, within only 15 min after light pulse, WCC transiently activates early light-responsive genes such as *albino-3*, *vivid*, and *frequency* triggering a long-term mechanism [26,27]. The transcription factor SUB-1 supports recruitment of -light-activated WCC to some promoters of its own target genes [25]. Moreover, many light-regulated genes are under delayed transcriptional control mediated by WCC and are expressed several hours after light induction [28,29,30]. WCC also acts as an inhibitor of *wc-2* transcription [26]. This inhibition is regulated via an unidentified repressor of *wc-2* transcription, which is expressed under the control of WCC [31]. After exposure to a light pulse, another factor that can modify WCC activity is Vivid, which is rapidly transcribed and translated. VIVID (VVD) is a small flavin PAS/LOV protein that binds blue-light photoreceptors [32,33]. It is involved in photoadaptation in *Neurospora*. Vivid inhibits WCC in a light-dependent fashion. The light-activated VVD acts as a competitive inhibitor of WCC homodimerization via its own LOV domain. It dampens the level of oscillating light-dependent transcripts, reverting them to the pre-induction phase (constant darkness) within 2 to 4 h after light exposure [34,35].

## 2. The *Neurospora crassa* Circadian Cycle

Circadian rhythms consist of daily physiological cycles driven by an endogenous biological clock and are entrained by zeitgebers, exogenous stimuli such as light, temperature, and nutrients [36]. These biological cycles are found in all living organisms from prokaryotes to mammals and are regulated by a molecular mechanism that is highly conserved across species [37]. Present at all levels of tissue organization, circadian rhythms are generated by individual cells and persist in the absence of external stimuli. Together with *Drosophila* and the mouse, *N. crassa* is a commonly used model system in the study of circadian rhythms [38,39]. The first documented *Neurospora* circadian rhythm was discovered in 1960 and was characterized as a sustained period of 22.5 h under constant conditions, roughly matching the Earth’s 24 h rotation cycle [40].

A variety of *Neurospora* mutants have been studied to better understand these rhythms, assigning them such rhythmic-inspired terms as *patch*, *clock* and *wrist watch*, *timex* and *band* [41,42,43,44]. Rhythmic spore formation can be easily monitored during growth as a pattern of thick conidiation “bands” alternating with thin “interbands” as the fungal mycelium advances across a solid agar surface and it is used as a phenotypic control of *Neurospora* circadian synchronization [45]. *Frq* is one of the genes transcribed after WCC activation [46]. It is the principal repressor involved in the negative loop of the circadian clock system [47]. Since *frq* is rapidly activated after multiple light pulses, FRQ also influences the light dependent gene transcription [48]. Light/Dark transition leads to a rapid disappearance of *frq* RNA and degradation of FRQ protein and WCC reactivation [49].

### Positive and Negative Feedback Loops in the Neurospora’s Circadian Clock

**Figure 2:**
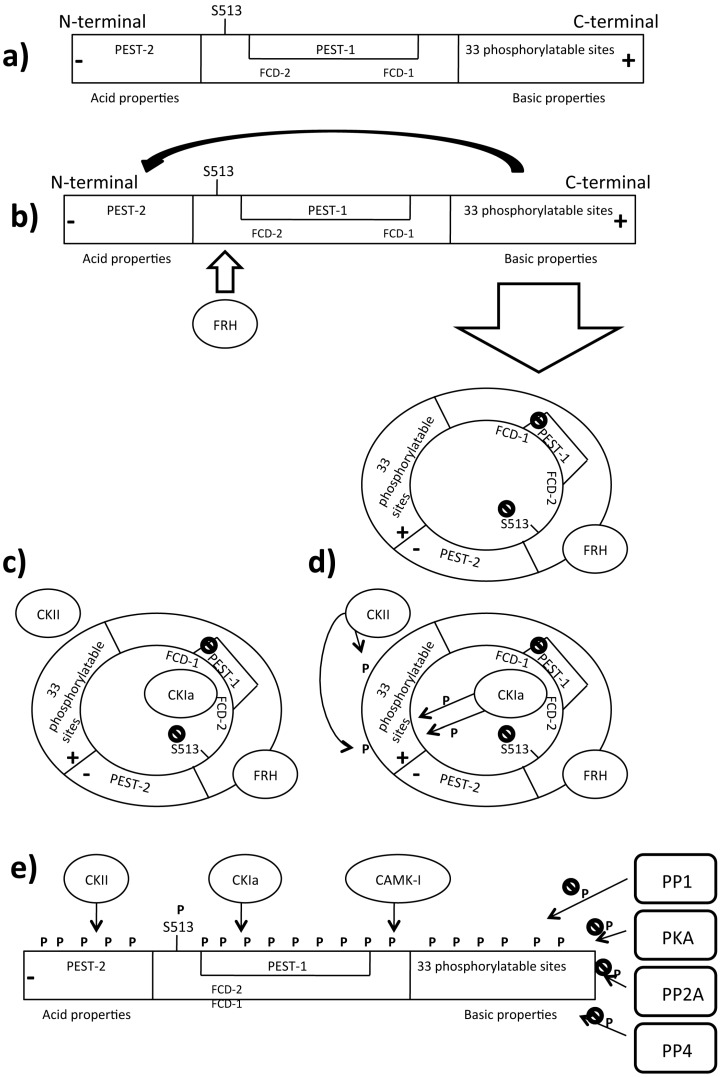
Phosphorylation related Frequency turnover. Legend: CAMK: Ca/CaM dependent kinase, CK: Casein kinase, E3: E3 ubiquitin ligase, FCD: Frequency/Casein kinase interaction domain, FRH: Frequency related helicase, FRQ: Frequency, FWD: F-box/WD40 repeat-containing protein, P: Phosphate group, PEST: P-E-S-T rich domain, PKA: Protein kinase A, PP: Protein phosphatase; (**a**) Domains that compose Frequency; (**b**) Newly synthesized FRQ recruits FRH that aids the correct fold of FRQ. The FRQ N-terminal domain interacts with the C-terminal domain, generating a closed structure that masks the PEST1 sequence and S513; (**c**) FRQ recruits the Casein kinase CKIa that binds the FCD sequences and physically interacts with FRQ. Other kinases such CKII are recruited on FRQ; (**d**) Casein kinases start to add phosphate groups to several FRQ domains; and (**e**) the addition of a negative phosphate groups abolishes the basic properties of the C-terminal domain, opening the closed structure. FRQ exposes its PEST sequences and the S513 residue that become targets of CKIa, CKII and CAMK1; at the same time many phosphatases contrast this activity, improving the FRA stability. When kinases’ activity overcomes the phosphatases’ activity, FRQ becomes highly phosphorylated and recruits FWD1 which is a substrate for E3 ubiquitinase (not shown in figure).

The current model describing the molecular mechanism driving the circadian rhythm in *N. crassa* consists of interlocked negative and positive feedback loops involving the clock genes *frq*, *wc-1*, and *wc-2* [46]. In constant darkness, WCC (D-WCC) acts on the positive loop and activates the transcription of the *frq* gene encoding the FRQ protein which binds its own C-box [50,51,52]. After *frq* gene transcription, *frq* mRNA reaches its peak during the subjective day, and FRQ protein peaks 4 to 6 h later. It was found that *frq* mRNA possesses another start codon located 100 codons downstream of the first ATG: alternative translation initiation produces a 135-kDa protein (889 amino acids) that lacks the normal N-terminal domain [53]. Short and long FRQ (SFRQ, LFRQ) are required for optimal clock function [54]. LFRQ is the core of the circadian negative loop [55]. FRQ contains some important domains among its sequence [56] (Figure 2a).

After importation into the nucleus, FRQ homodimerizes via the N-terminal coiled coil domain and forms a functional homodimer [56]. LFRQ is also involved in temperature compensation of the clock [57,58]. Throughout this cycle, FRQ interacts with several proteins including FRH homologous to the yeast RNA-binding protein Dob1p/Mtr4p [59]. FRH associates with and stabilizes FRQ in an ATP-independent manner and it is essential for correct FRQ folding [60] (Figure 2b). In *Neurospora* the entire pool of FRQ is in complex with FRH; down regulation of *frh* expression abolishes circadian rhythmicity, resulting in high *frq* RNA levels. FRH binds the FRQ dimer in a stoichiometric manner to form a complex known as FCC; this complex acts as a part of the negative loop of the clock, inhibiting activity of the D-WCC and *frq* gene expression [61]. During this phase, FCC acts as a platform that recruits numerous kinases that will phosphorylate WCC and lead it to the degradation pathway (see Section 3.8). When FRQ levels drop below a certain threshold in the subjective late night, D-WCC is no longer inhibited by FRQ, and *frq* transcription is reactivated to start a new cycle [62]. Moreover, FRH acts non-enzymatically to support the intrinsically disordered FRQ [63]. This succession of positive and negative loops results in the circadian rhythmic oscillation of *frq* mRNA and protein, which is critical for the normal circadian behavior of an organism [64]. Moreover, FRH overexpression promotes WC-1 accumulation, confirming that FRH, together with FRQ, plays a role in stabilizing WC-1 [60]. Indeed, FRH-FRQ interaction is essential for maintaining correct WC protein levels in the positive feedback loops. Besides its role in repressing its transcription, FRQ also positively regulates the levels of both WC-1 and WC-2 through different mechanisms [65]. In the cytoplasm, FRQ acts as a positive factor by supporting WC-1 protein accumulation, leading to the formation of the WCC via an unknown mechanism [66]. The C-terminal part of FRQ is fundamental for its cytosolic localization [67]. This suggests that there are two different kinds of FRQ-mediated regulation of the circadian clock [68]. As said before, another light pulse can induce light-dependent *frq* mRNA to reset the clock when the *frq* level decreases. The Light/Dark transition is a highly reliable method for resetting circadian rhythm. Although the WCC/FRQ complex represents the core of the circadian system in *Neurospora*, other factors are also involved in this regulatory process.

## 3. Protein Posttranslational Modifications (PTMs) during the Circadian Clock, and Light Signal Transduction

Posttranslational modifications (PTMs, e.g., phosphorylation and acetylation) alter protein behavior in response to conditional changes and regulate protein activity through different mechanisms, including protein-protein interactions, protein localization, degradation, and cleavage or allosteric regulation of enzyme activity [69,70,71]. They can activate or deactivate other regulators, thereby changing the PTM status of downstream proteins.

The basis of circadian rhythm generation is similar in all eukaryotes. The CLOCK/CYCLE heterodimer in *Drosophila* and CLOCK/BMAL1 in mammals are transcriptional activators, while PER/TIM in *Drosophila* and PER2/CRY in mammals are negative transcriptional factors [72]. Many PTMs regulate the activity of each circadian factor [73]. Phosphorylation is the most important PTM that regulates circadian rhythms [74]. The *Drosophila double time* (*Dbt*) gene, which encodes casein kinase 1 (CK1), was the first posttranslational modifier to be linked to the control of circadian rhythmicity by phosphorylating the Period (PER) transcription factor and influencing its own stability and subcellular localization [75]. DBT phosphorylates PER at several sites in a stepwise fashion and mediates its degradation by recruiting a conserved F-box/WD40-repeat protein (Slimb) [76]. This complex drives PER to the ubiquitin/proteasome pathway [77]. As described in *Drosophila*, PER2 in mammals is similarly regulated by phosphorylation [78]. Altered phosphorylation of human PER2 is responsible for the familial advanced sleep phase syndrome (FASP) caused by a mutation (S662G/) in the amino acid sequence [79]. Phosphorylation of the mammalian Clock, BMAL and the photoreceptor Cryptochrome 1 (CRY1) is widely described and associated with their stability and activity [80,81,82,83,84,85].

Acetylation, another important PTM in circadian and non-circadian systems, influences protein stability, protein-protein interactions, as well as translocation inside and outside the nucleus [86]. The transcriptional factor BMAL1 is acetylated by Clock and deacetylated by SIRT1 [87,88]. BMAL acetylation modifies its activity and ability to recruit the PER2/CRY heterodimer during the negative feedback loop in the mouse circadian rhythm [88]. Deacetylation of PER2 mediated by SIRT1 promotes its degradation [89]. The components of the *Neurospora* circadian clock do not show strong structural homology with those of *Drosophila*, mammalian or human clocks, though they share many common functional features. Recent data suggest complex posttranslational regulation in the *Neurospora* clockwork through a molecular mechanism highly similar to higher eukaryotes. Here we outline the principal PTMs that regulate the non-histone proteins FRQ, WC-1 and WC-2 and similar modifications at level of chromatin.

### 3.1. Faces and Timing of Frequency (FRQ) Phosphorylation

FRQ is progressively phosphorylated during the day. Several evidences support that FRQ phosphorylation is related to its daily turnover [52,90]. First, *in vivo* phosphorylation is blocked after treatment with 6-DMAP, a general kinase inhibitor, and the total FRQ amount is increased, thus demonstrating the role of phosphorylation in FRQ stability [49]. Moreover, phosphorylation is essential for maintaining “in phase” the *Neurospora* circadian rhythm, as observed in strains treated with 6-DMAP in which circadian conidiation was aberrant [49]. Second, by systematic mutagenesis of the FRQ open reading frame (ORF), S513 has been identified among different putative phosphorylation sites as the most important. Elimination of S513 leads to a dramatic reduction in the rate of FRQ degradation and a significantly increased period caused by an increased stability of FRQ [49,91,92,93,94,95,96]. Third, over 75 sites have been identified by quantitative proteomics using nanoscale micro capillary liquid chromatography-mass spectrometry/mass spectrometry (LC-MS/MS or LC tandem MS) and analyzed by stable isotope labeling with amino acids in cell culture (SILAC) quantification [93]. These sites are mapped along the entire FRQ amino acid sequence. FRQ can be subdivided into three main regions: the acidic *C*-terminal domain, the central domain, and the N-terminal domain that shows basic properties [94]. FRQ shows two PEST-like domains containing proline (P), glutamic acid (E), serine (S) and threonine (T) rich domain: PEST-1 in the central domain and PEST-2 in the N-terminal domain (Figure 2a). These domains are highly phosphorylated. Interestingly, deletion of the PEST-1 domain in FRQ results in an arrhythmic conidiation phenotype. Deletions of PEST-1 or mutations of S513 generate a very stable form of the FRQ protein, suggesting that loss of oscillating FRQ phosphorylation affects *Neurospora* circadian behavior. Deletion of PEST-2 does not reduce FRQ expression, but it is required for WC-1 stability. Some experimental evidence suggests that WC-1 accumulation is regulated by phosphorylation of FRQ at S513, S885, and S887. Mutation S885, 887 can affect the positive limb of the interconnected feedback loops, while deletion of the entire PEST-2 domain causes a more severe functional defect. PEST domain is involved in protein turnover in several organisms and seems to have the same function in FRQ [95]. The kinetics of FRQ oscillation is spatially and temporally coordinated by phosphorylation [96]. After the light/dark shift that leads *Neurospora* circadian entrainment (time point 0), a high amount of FRQ is hypophosphorylated in constant darkness (DD) up to 14 h after light synchronization (DD14, corresponding to subjective morning). A low amount of FRQ protein is highly phosphorylated 24 h after light synchronization (DD24, corresponding to subjective night). This means that at DD14 the total amount of FRQ is higher than the total amount at DD24, suggesting that phosphorylation is associated with the degradation pathway [23]. Newly synthesized hypophosphorylated FRQ contains a nuclear localization signal (NLS) and is trapped in the nucleus more efficiently than hyperphosphorylated species [56,66]. Despite the presence of a NLS, only a small fraction of FRQ is localized in the nucleus and the majority of the protein accumulates in the cytosol [23,96]. FRQ phosphorylation inhibits nuclear import, and retains the proteins in the cytoplasm where it acts as a positive factor by supporting the WC-1 protein accumulation, as said before. Since phosphorylated FRQ exert diverse functions such as support of WCC accumulation in the cytosol and inactivation of WCC activity into the nucleus, it indirectly influences the daily oscillation of hundreds of genes that are under the control of the WCC as well.

Recent observations provided evidence that FRH is critical for the maintenance of FRQ phosphorylation profile and stability [67]. Loss of FRQ-FRH interactions, or down-regulation of FRH, promotes the hypophosphorylated form of the FRQ:FRQ altering the normal circadian pathway [97]. Many kinases are involved in FRQ regulation. In the following paragraph, we will describe the principal kinases involved in the full length FRQ phosphorylation.

### 3.2. Kinases Targeting FRQ

#### 3.2.1. CKIa and CKIb

Two putative kinases involved in FRQ phosphorylation are CKIa and CKIb, which are homologous to human kinase 1 epsilon [98,99]. CKIa co-immunoprecipitates with FRQ in both dark and light conditions, suggesting that it is a member of the FRQ complex throughout the entire circadian cycle. It shows a substantial preference for the highly phosphorylated FRQ form [48]. Prevention of the interaction between FRQ and CKIa leads to a hypophosphorylated state of FRQ. FRQ is hypophosphorylated and shows slow degradation in the *CKIa*^L^ mutant strain in which the activity of CKIa is abolished, confirming that FRQ is a target of CKIa *in vivo*. Finally, the progression timing of FRQ phosphorylation is delayed in this mutant [98].

FRH is also known to associate with FRQ, stabilizing it in an ATP-independent manner. The ATPase of FRH attenuates the kinetics of CKIa-dependent hyperphosphorylation of FRQ, suggesting that phosphorylation regulates the entire FCC complex [100]. CKIa interacts with FRQ by two sequences, called FCD2 (aa 488 to aa 495) and FCD1 (162 amino acids downstream of FCD2) located in the central domain of FRQ named FCD. The FCD region (aa 470 to aa 532) also includes the PEST-1 domain. Deletion of one of the FCD domains totally abolishes CKIa binding on FRQ, indicating that a high-phosphorylated form of FRQ is necessary for its binding [48,98]. *In vitro* experiments demonstrated that both CKIa/CKIb target PEST-2; however, tandem MS analyses were unable to recognize highly phosphorylated sites within this region. Only CKIa targets PEST-1. The FRQ N-terminal domain contains 33 residues that can be phosphorylated by various kinases. In particular, CKIa and CKIb have been suggested for this role, though CKIb is onlyable to phosphorylate the N-terminal domain *in vitro* [101]. Figure 2c–e shows where CKIa is mapped along FRQ sequence.

#### 3.2.2. CKII

Another kinase, the *Neurospora* casein kinase II (CKII), was isolated and characterized starting from *camk-1* mutant complementation [102,103]. CKII is a holoenzyme composed of three subunits: CKA, CKB1, and CKB2. CKA is the catalytic subunit; CKB1 and CKB2 are the regulatory elements. Phosphorylation of FRQ by CKII is important in regulating the stability of FRQ. The FRQ phosphorylation pattern was significantly altered in *cka^RIP^* mutants in DD and LL phases and the levels of FRQ were higher in the mutants than in the wild-type strains. Slower FRQ degradation was observed in the Light/Dark transition. In the *N. crassa* wild-type strain, FRQ was rapidly degraded after the Light Dark (LD) with 40% FRQ left after 8 h in the dark. In contrast, FRQ degradation rate was significantly slower in a *cka^RIP^* strain after the LD transition (80%) of FRQ was still present in the mutant after 8 h in the dark). Again, the increased amount of FRQ found in the mutant is consistent with the fact that phosphorylation of FRQ triggers its own degradation [49]. Phosphorylation of FRQ by CKII plays important roles in regulating the formation of the FRQ:WC complex and the closing the circadian negative feedback loop. FRQ nuclear localization was unchanged in *cka^RIP^* mutant strains but CoIP assay demonstrated that a large amount of FRQ coprecipitated with WC-1 in the mutant strain, suggesting that the hypophosphorylated FRQ in the *cka^RIP^* mutant stabilizes and promotes the formation of the FRQ:WC complex. These data indicate that phosphorylation of FRQ by CKII decreases FRQ’s ability to interact with the WC proteins. The circadian negative feedback loop was impaired in the CKII mutant and FRQ failed to inhibit the transcriptional activation activity of the WC complex effectively in the CKII mutant even if it strongly interacts with WCC. This means that although the interaction between FRQ and the WCs is necessary for the closing of the circadian negative feedback loop the interaction alone is not sufficient for FRQ to act to repress the transcriptional activation of *frq* by the WC proteins [65,104]. The molecular phenotypes of CKII mutants are very similar to those of the *frq null* strain. We can conclude that *Neurospora* CKII is an important component of the *Neurospora* circadian system. Figure 2c–e shows where CKII is mapped along FRQ sequence.

#### 3.2.3. CAMK1

Nakashima and colleagues have shown that the circadian and light dependent conidiation rhythm of *Neurospora* can be phase-shifted by CaM antagonists [105,106]. This finding and the identification of a 50 kDA Ca/CaM-dependent kinase CAMK1 as one of the kinases targeting FRQ [91] suggested the involvement of CaM kinase in FRQ activity. It is very similar to other eukaryotic Ca/CaM-dependent kinases, with a highly conserved catalytic domain. *In vivo* and *in vitro* assays have demonstrated that CAMK1 is responsible for FRQ phosphorylation. The phosphorylation activity requires both Ca^2+^ and CaM. A chelator of Ca^2+^ can also inhibit the phosphorylation of the endogenous FRQ in *Neurospora* extracts. CAMK-1 accounts for near half of the FRQ phosphorylation, including the region aa 501–519, which contains the three known functionally important phosphorylation sites (T501, S513, S519). The circadian clock is affected in the *camk-1* KO mutant, in which the running phase of conidiation showed a modest delay as compared with the wild-type strain. The phenotypes of the *camk-1* null strain suggest that it plays an important role in growth and development. The phase response to light was also altered in the *camk-1* mutant. All together these data evidenced the importance of the FRQ phosphorylation in the regulation of the *Neurospora* circadian clock and light signal transduction. The mild clock phenotype and the significant FRQ kinase activity in the *camk-1* KO strains suggested that FRQ might be phosphorylated also by other kinases. Figure 2e shows where CAMK is mapped along FRQ sequence.

### 3.3. FRQ Stabilization

#### 3.3.1. PKA

Unlike the kinases previously described, which promote FRQ degradation, protein kinase a 1 (PKA) stabilizes FRQ protein [107] (Figure 2e). In strains with high PKA activity, like *mcb* strains, *frq* mRNA levels are extremely low, but FRQ protein is at an intermediate level of abundance in DD and it is more stable than in the wt strain. In addition, FRQ is hypophosphorylated in DD in the *pkac-1* KO strains [107]. These data reveal that the PKA-mediated FRQ phosphorylation in *Neurospora* has effects similar to hPER2 phosphorylation at the FASPS sites in humans [108,109]. The *Neurospora* PKA can directly phosphorylate FRQ *in vitro* and FRQ phosphorylation mediated by PKA stabilizes the protein but PKA does not associate tightly with FRQ and the mechanism is still unclear to date.

How does PKA-mediated phosphorylation of FRQ inhibits FRQ own degradation pathway? It has been proposed that this phosphorylation probably either alters the FRQ structure conformation or inhibits its phosphorylation by CK-1a and CKII in a way that it cannot be efficiently ubiquitinated and degraded. A fairly similar mechanism was observed in mammals. In the mammalian WNT signaling pathway, for example, phosphorylation of β-catenin by PKA is known to inhibit the ubiquitination of β-catenin promoted by CKI and glycogen Synthetase kinase 3 β (GSK-3 β) phosphorylation [110,111].

#### 3.3.2. PP1, PP2 and PP4

A dephosphorylating pathway that can counteract the increasing phosphorylating level of FRQ requires phosphatase enzymes. PP1 and PP2a were identified in *N. crassa* and suggested as regulators of the *Neurospora* circadian clock [112]. The *ppp-1^RIP^*mutant affects FRQ dephosphorylation, compromising the protein’s normal degradation rate, which is faster than in the wild type and it also affects *Neurospora* circadian entrainment. Disruption of the catalytic subunit RGB-1 of PP2a (*rgb-1^RIP^* mutant) affects the normal phosphorylation cycle and normal circadian conidiation in *N. crassa* as well. This suggests that FRQ is an *in vivo* target of PP1 and PP2a, even though only PP1 is really able to affect FRQ protein stability. Low FRQ protein and *frq* mRNA levels were observed in the *rgb-1^RIP^* strain, differently from what was observed in the *CKII* mutant, suggesting that PP2A activity counteracts CKII activity and probably prevents closing of the negative feedback loop (Figure 2e).

Another phosphatase identified in *N. crassa* is PP4 (Figure 2e), a homolog of the human catalytic subunit of protein phosphatase 4 [113,114]. Mutant strains that abolished PP4 activity showed a deregulation of circadian rhythm period and amplitude. PP4 physically interacts with FRQ, which is hyperphosphorylated and increases its degradation rate in a *pp4* KO strain. These data suggest that PP1 and PP2a and PP4 all antagonize the effects of the kinases and stabilize FRQ protein. Table 1 lists the proteins implicated in FRQ turnover.

**Table 1:** Proteins interacting with FRQ.

Protein	Target	Activity	Effects	Other Information
FRH	FRQ	Promotes FRQ conformational changes	Ensures proper folding of FRQ	NA
			Masks some residues to CKIa phosphorylation	
	*frq* mRNA	FRH physically interacts with *frq* mRNA	Exosomal degradation of mRNA	
CKIa	FRQ N-terminal domain	Phosphorylation	Conformational changes	Recruited on FRQ FCD domains
	FRQ PEST-1 domain	Phosphorylation	FWD-1 recruitment	NA
	FRQ S513	Phosphorylation	FWD-1 recruitment	NA
	FRQ N-terminal domain	Phosphorylation	FWD-1 recruitment	NA
	FRQ Central domain	Phosphorylation	FWD-1 recruitment	NA
CKII	FRQ N-terminal domain	Phosphorylation	Conformational changes	Recruited on FRQ FCD domains
	FRQ PEST-1 domain	Phosphorylation	FWD-1 recruitment	NA
	FRQ S513	Phosphorylation	FWD-1 recruitment	NA
	FRQ N-terminal domain	Phosphorylation	FWD-1 recruitment	NA
	FRQ Central domain	Phosphorylation	FWD-1 recruitment	NA
CAMK-1	FRQ N-terminal domain	Phosphorylation	FWD-1 recruitment	NA
	FRQ PEST-1 domain	Phosphorylation	FWD-1 recruitment	NA
	FRQ S513	Phosphorylation	FWD-1 recruitment	NA
	FRQ N-terminal domain	Phosphorylation	FWD-1 recruitment	NA
CKIb	FRQ N-terminal domain	Phosphorylation	Conformational changes	Interaction with N-terminal domain demonstrated only *in vitro*
PP1	FRQ PEST-1 domain	Dephosphorylation	Contrasting phosphatase action–FRQ stabilization	NA
	FRQ S513	Dephosphorylation	Contrasting phosphatase action–FRQ stabilization	NA
	FRQ N-terminal domain	Dephosphorylation	Contrasting phosphatase action–FRQ stabilization	NA
	FRQ Central domain	Dephosphorylation	Contrasting phosphatase action–FRQ stabilization	NA
PP2A	FRQ PEST-1 domain	Dephosphorylation	Contrasting phosphatase action–FRQ stabilization	NA
	FRQ S513	Dephosphorylation	Contrasting phosphatase action–FRQ stabilization	NA
	FRQ N-terminal domain	Dephosphorylation	Contrasting phosphatase action–FRQ stabilization	NA
	FRQ Central domain	Dephosphorylation	Contrasting phosphatase action–FRQ stabilization	NA
PP4	FRQ PEST-1 domain	Dephosphorylation	Contrasting phosphatase action–FRQ stabilization	NA
	FRQ S513	Dephosphorylation	Contrasting phosphatase action–FRQ stabilization	NA
	FRQ N-terminal domain	Dephosphorylation	Contrasting phosphatase action–FRQ stabilization	NA
	FRQ Central domain	Dephosphorylation	Contrasting phosphatase action–FRQ stabilization	NA
FWD-1	Hyperphosphorylated FRQ	Physical interaction with FRQ	SCF-type ubiquitin E3 ligase recruitment	NA
SCF-type ubiquitin E3 ligase	Hyperphosphorylated FRQ	Ubiquitination of FRQ	FRQ proteasomal degradation	NA

### 3.4. Molecular Mechanisms of FRQ Phosphorylation and Proteasomal Degradation

Actually there are described at least 113 phosphorylated sites identified in FRQ [93,101]. MS analyses showed that the full-length FRQ phosphorylation is favored when FRQ is preliminarily phosphorylated in some specific sites, suggesting that it is regulated by a specific mechanism for phosphorylation. One explanation of this process is that newly synthesized, hypophosphorylated FRQ adopts preferentially the closed conformation supported by electrostatic interactions of the positively charged N-terminal domain with the negatively charged remainder portion of the protein [115]. Early phosphorylation of some sites in the C-Term region and middle portion could assist to strengthen the closed state that allows stabilization of FRQ.

Subsequently CKIa and CKII start to phosphorylate the N-terminal of FRQ, masking the basical properties of the N-terminal domain. As a result, N-terminal domain can no longer interact with the C-terminal acid domain. This event causes a conformational change in FRQ that exposes the S513 and the PEST-1 sequence of the central domain. Then CKIa, CKII, and CAMK-1 phosphorylate the PEST sequences, the S513 residue, and many other residues dislocated along the entire protein, except for the C-terminal domain, which is never phosphorylated. This means that the circadian oscillation of FRQ phosphorylation is due to multiple reactions throughout the FRQ structure and in the spatial and temporal combination of these kinases [116]. The FRQ phosphorylation pathway is illustrated in Figure 2. The combination of these phosphorylation processes leads FRQ to the degradation pathway. Some findings indicate that hyperphosphorylated FRQ is degraded by proteasome. First, ophiobolin A, a well-known calmoduline inhibitor tested to identify CAMK involvement in FRQ phosphorylation, promotes protein ubiquitination and the formation of higher molecular weight conjugates of FRQ *in vivo* [117]; Second, disruption of the *fwd1* gene (homolog of the *Drosophila* Slimb protein), which encodes a protein containing F-box and VD4causes hyperphosphorylation of FRQ with a pattern different from that observed in wild-type strains [118]. FWD, a member of the SCF-type ubiquitin ligase complex, seems to directly ubiquinate FRQ. Hence, FWD mutation decreases the FRQ degradation rate because the protein appears to be more stable than in the wild-type strain; Third, the accumulation of FRQ in the *fwd-1* mutant strain is associated with dysfunctions in circadian oscillation though the Dark/Light transition still occurs; Fourth, FRQ physically interacts with FWD-1 *in vivo*, driving FRQ to the degradation pathway, as observed in the *fwd-1 mutant* that was still able to bind FRQ but lacked the F-box domain involved in FRQ degradation. In summary, these data indicate that FRQ phosphorylation promotes FRQ degradation through the ubiquitin-proteasome pathway mediated by ubiquitin E3 ligase (Figure 2f).

### 3.5. White Collar Complex (WCC) Light Dependent Phosphorylation

**Figure 3:**
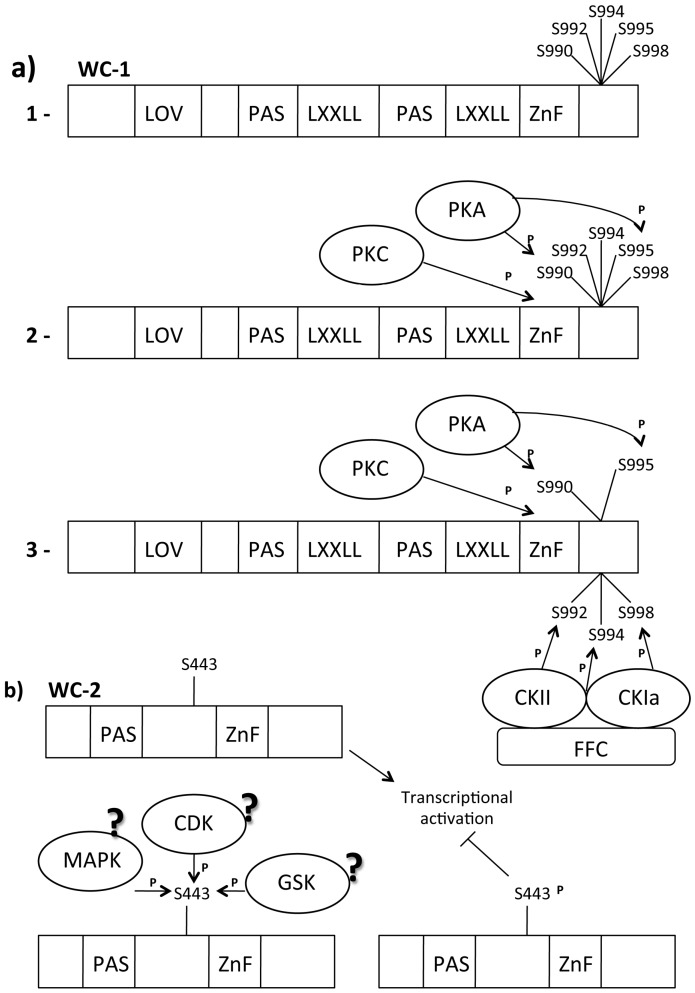
WC-1/WC-2 post translation modification (PTM) Modifications. Legend: ?: Supposed, CDK: Cyclin dependent kinase, CK: Casein kinase, FRQ: Frequency, GSK: Glycogen Synthetase kinase, LXXLL: WC-1/NGF1 interaction domains, LOV domain: Light-Oxygen-Voltage domain, MAPK: MAP activated protein kinase, P: Phosphate group, PAS domain: Per Arnt Sim domain, dimerization domain, PKA: Protein kinase, AS443: Phosphorylatable Serine residue, S990–992–994–995–998: Phosphorylatable Serine residues, Zn finger: Zinc finger, WC-1/DNA binding domain. (**a**): (**1**) Important WC1 domains; (**2**) PKA is an FRQ-independent kinase that phosphorylates the WC-1 S990 and S995; (**3**) Phosphorylation of S990 and S995 is a signal for CKII and CKIa (kinase recruited by FRQ) to phosphorylate S992, S994 and S998. This phosphorylation destabilizes WC-1, generating a transcriptional repression of light-activated genes; (**b**) TheWC2 active state is non-phosphorylated. When S433 became phosphorylated the WCC activity results reduced.

Evidence suggests that the light-dependent WCC pathway is influenced by protein phosphorylation [119,120]. It has been demonstrated that WC-1 becomes highly phosphorylated 15 min after multiple light pulses and that the phosphorylation level decreases to the same levels as in dark after 2 h [121,122]. Functional WC-2 is not required for PTM of WC-1 but it could influence the degree of PTM and the stability of WC-1. Light-dependent WC-1 phosphorylation oscillation correlates with the expression of light-dependent gene (e.g., *al-3*). As observed for FRQ, WC-1 phosphorylation is also coupled with its own degradation. Pharmacological studies and *in vitro* phosphorylation assays have suggested that protein kinase C (PKC) may phosphorylate WC-1 [123] (Figure 3a-2).

*In vivo* and *in vitro* experiments have confirmed that PKC regulates light responses affecting light-dependent WC-1 protein stability [124,125]. PKC seems to be able to phosphorylate the WC-1 zinc-finger domain. The zinc-finger domain is important for the function of WC-1 in the dark [26]. PKC physically interacts with WC-1 in dark conditions, but this interaction is no longer detectable after 30 min of light exposure when WC-1 is hyperphosphorylated. This suggests that light-mediated WC-1 hyperphosphorylation affects PKC/WC-1 interaction. Phosphorylation of WC-2 appears to be more stable over time after light exposure [122] and shows strong oscillation when analyzed by two-dimensional electrophoresis [126]. The cytoplasmic WC-2 protein is not modified in response to light, suggesting that light-specific phosphorylation is limited to nuclear pool of WC-2 [122]. Finally, it has been demonstrated that WC-2 light-specific phosphorylation depends on a functional WC-1. The translocation of WC-1 and WC-2 into the nucleus is independent of light-specific phosphorylation, unlike that observed for FRQ phosphorylation [23]. MS analyses identified S443 as the phosphorylation target in WC-2 protein [127] (Figure 3b).

S433 plays a role in the regulation of WCC activity but it does not affect WCC turnover according to mutagenesis experiments. The activity and the stability of WCC were increased and the timing of photoadaptation was anticipated (probably due to more rapid accumulation of the blue-light photoreceptor VVD) in the *s433a* strain, a WC-2 variant in which S443 is mutated in A, thus preventing WC-2 phosphorylation. In contrast, WCC was less active in the *s433d* strain in which mutation of Sto Emimics the phosphorylated state of WC-2. The phosphorylation site S433 is located directly upstream of a prolyl residue, suggesting that it could be target of serine/threonine kinases such as MAPK-activated protein kinase (MAPK) and Cyclin-Dependent Kinase (CDK) [128]. Table 2 lists the kinases that target the White Collar Complex.

**Table 2:** Kinases targeting the White Collar Complex.

Protein Name	Protein Target	AA Target	Activity	Effects	Other Information	Other Information
PKA	WC-1	S990 S995	Phosphorylation	Inhibition WCC activity Putative Priming kinase	FRQ independent kinase	Interaction experimentally proven
PKC	WC-1	Zn Finger	Phosphorylation	Decreased WC-1 stability	NA	Interaction experimentally proven
CKIa	WC-1	S992 S994 S998	Phosphorylation	Decreased WC-1 activity	Kinases recruited by FRQ	Interaction experimentally proven
CKII	WC-1	S992 S994 S998	Phosphorylation	Decreased WC-1 activity	Kinases recruited by FRQ	Interaction experimentally proven
GSK	WC-1		Phosphorylation		NA	Interaction experimentally proven
Unknown	WC-2	S443	Phosphorylation	Decreased WCC activity	NA	NA

### 3.6. White Collar 1 (WC-1) Light Dependent Acetylation

Recent evidence suggests that WC-1 can be acetylated *in vitro* and *in vivo* along the entire sequence [129]. Though the role of WC-1 acetylation is not clear, this PTM seems respond to light signals. *Neurospora* Gcn Five-1, (the histone acetyl transferase homologous to the Yeast Gcn5p and Human GCN5) could be involved in acetylation of WC-1 as well as histone H3 of light induced gene promoters [130]. However, analysis of the *nfg1^RIP^* mutant did not confirm this hypothesis because WC-1 was still acetylated in this mutant strain.

### 3.7. The FRQ-Independent WCC Circadian Phosphorylation

MS/MS analyses identified five phosphorylated Sin WC-1 (S990, S992, S994, S995, S998), which are phosphorylated *in vivo* and localize in a region immediately downstream of the zinc-finger domain [131] (Figure 3a-1). Biochemical analyses have demonstrated that these five phosphorylation sites are not involved in the light-induced hyperphosphorylation of WC-1. However, phosphorylation of these sites appears to be necessary for maintaining WC-1 steady state levels, as observed in strains in which these serine were mutated. Mutations at these sites affect normal circadian oscillation in *N. crassa* though they do not affect light-dependent responses. They might be involved in the positive limb of the circadian negative feedback loop [111]. The amount of WC-1 was found to determine the expression of *frq* in the dark in a wild-type strain; in the mutant with all five sites mutated, the *frq* mRNA level was slightly higher, suggesting that these phosphorylation events negatively regulate the transcriptional activation of WC-1 in the dark. S990 was the first of the five phosphorylated Serine mentioned above to be phosphorylated. Several experiments indicate protein kinase A (PKA) for this role (Figure 3a-1): both WC proteins are hypophosphorylated in the *pka^C^* mutant strain, they physically interact with PKA and are phosphorylated by this kinase; PKA associates with WCC but not with FRQ, suggesting that phosphorylation of WCs by PKA is independent of FRQ; *in vivo* experiments demonstrated that PKC phosphorylates WC-1 in the region near the DNA binding domain and that this phosphorylation stabilizes the WC-1 protein; phosphorylation of WCs by PKA strongly inhibits WCC activity, resulting in the inhibition of *frq* transcription. Phosphorylation occurs upstream from CK-1a and CKII during WCC inhibition. Finally, PKA and its regulatory subunit PKAR are essential for normal circadian rhythm because WC-1 is less stable in a PKA mutant strain and the circadian transcription of clock-controlled genes is compromised. These data suggest that PKA can act as a priming kinase on WC-1. Bioinformatics analyses have shown that the sequence context around residues 990 (KSNSP) might be a direct target of PKA, whereas S995 (SHSSP) is not a typical PKA site (R-X-S/T or R-R/K-X-S) [132].

The FRQ-independent phosphorylation of S990 and S995 can convert the other sites into CKIa and CKII targets [107]. Phosphorylation of S990 significantly enhanced the ability of CK-1a to phosphorylate this region. This means that phosphorylation of S990 mediates the recruitment of the other kinases and that the subsequent phosphorylation events are mediated by a priming kinase. Recently, GSK entered in the group of the circadian clock regulators of *N. crassa*; GSK physically interacts with the WCC, phosphorylating both subunits with high specificity in a complex protein environment *in vitro* [133]. GSK also influences WC-1 protein stability, as observed in strains in which GSK (Glycogen Synthetase Kinase) activity was reduced, and it was coupled with an increased amount of WC-1. Since GSK affects the free-running period of *Neurospora*, the kinase must act on the dark form of the WCC.

### 3.8. The FRQ-Dependent WCC Circadian Phosphorylation

In the nucleus, the WCC is present in excess over FRQ. Consequently, FRQ can neutralize only a very small fraction of WCC by binding stoichiometrically the complex and by regulating WCC phosphorylation [126]. An analysis carried out in different *frq* mutants (*frq*^10^, *frq*^9^) showed the typical loss of electrophoretic mobility of phosphorylated proteins for WC-1 and WC-2. Since the *frq*^9^ mutant produces a truncated FRQ protein lacking the C-terminal domain, this domain seems to be essential for WC-1 phosphorylation, as well as for shuttling of FRQ into the nucleus. FRQ-dependent phosphorylation leads to WCC inactivation [62]. This means that the phosphorylation level of the WCC during the circadian cycle correlates with the function of WCC as a transcription activator. Hypophosphorylated WCC efficiently binds the C-box of *frq* promoter, while hyperphosphorylated WCC binds the C-box with reduced affinity and does not efficiently activate the transcription of target genes.

How does FRQ influence WCC phosphorylation during daily circadian oscillation? FRQ physically interacts with CKIa and it is cyclically phosphorylated by this kinase (Figure 2c,d). It also forms a complex with WCC in the nucleus, suggesting that WCC could be target of CKIa. Immunoprecipitation experiments demonstrated the physical interaction between WC-1 and CKIa and this binding is FRQ-mediated. Disruption of the interaction between FRQ and CKIa also affects the binding between WC-1 and CKIa [98]. Functional mutation of CKIa (*ck-1a*^L^ strain) confirmed the involvement of this kinase in WCC phosphorylation.

FRQ/CKIa interaction is regulated by FRH [106] (Figure 2b,c); FRH with FRQ forms the FFC complex, which inhibits WCC activity [100]. This means that FRH is also important for WCC circadian phosphorylation. Moreover, WCC is also phosphorylated by CKII [98]. These results suggest that FRQ acts as a kinase substrate-recruiting subunit to mediate the phosphorylation of WCC by recruitment of CKIa and CKII. In turn, WCC phosphorylation affects its ability to be recruited at the C-box and LRE elements of the promoter sequences of regulated genes. WC-1 rhythmically binds the *frq* C-box, but this binding is enhanced in the *ck-1a*^L^ strain, confirming that a hypophosphorylated form of the WCC is required for its transcriptional activity [98].

The daily increase in WC-1 protein abundance seems to be correlated with the hyperphosphorylated form of FRQ. Accumulating data suggest that FRQ modulates the stability of total WC-1 protein, promoting its own accumulation and turnover [68]. Physical interaction between FRQ and WC-1 is essential for this process, and it has been suggested that FRQ-dependent clearance of WC-1 from the nucleus is a closure mechanism of the negative feedback loop mediated by oscillation in the FRQ: WCC ratio [134]. Translocated WC-1 can be reactivated in the cytoplasm in an FRQ-mediated manner [23,66]. Though it is not clear how FRQ can reactivate WC-1, it probably mediates the formation of the WCC.

## 4. Chromatin Epigenetic Modifications Involved in Circadian Rhythms and Light Signal Transduction

Chromatin is a not a simple packaging system of eukaryotic DNA but a more or less condensed nucleo-protein structure, modulated in time and space and associated with on/off states of transcriptional activity. In fact, condensed chromatin presents an obstacle for the double helix binding of transcription factors, resulting in a general inhibition of gene expression [135,136]. Chromatin consists of positively charged tails of histone proteins interacting with the negatively charged DNA phosphate backbone. Chromatin remodeling derives from the activity of histone modifiers affecting histone-DNA interaction by altering the charge of the histone tail [137] or/and from the activity of enzymes altering the DNA topology.

The modifiers can methylate, acetylate or phosphorylate histones, and the combination of these modifications is coupled with the state of chromatin [138,139]. Transcriptional activation is generally preceded or accompanied by local remodeling of chromatin structure. Since the histone tail alters the nucleosome structure via a number of active and inactive histone marks, inducing either a condensed silenced state or a decondensed active state of chromatin, the term “histone code” was coined to describe this process [140].

In mammals and plants, rhythmic changes in chromatin structure have been correlated with changes in the transcriptional activity of clock-associated *loci* and light responses. In mammals, acetylation of histone H3 at *Per1*, *Per2*, and *Cry* (genes encoding negative transcription factors involved in the mammal circadian clock) and acetylation of H4 at *Per1* coincide with the peak during the transcriptional activation phase [141]. In contrast, histone H3 associated with *Per1* and *Per2* becomes methylated at K27 or K79 during the repressive phase [142,143]. A transient burst of histone H3 phosphorylation on residue S10 (H3S10) has been observed in mammalian hypothalamic suprachiasmatic nuclei following multiple light pulses [144]. Recently, mixed-lineage leukemia (MLL1) lysine methyltransferase (KMT2A) has been shown to associate with the circadian core CLOCK-BMAL and is required for the rhythmic expression of clock genes [145]. In mammals and plants, Jumonji domain-containing protein 5 (JMJD5) lysine dimethyltransferase (KDM8) is rhythmically expressed and is required for normal circadian rhythms [146,147]. In plants, the circadian clock regulates the pattern of H3 acetylation of the *Timing of Cab 1* gene (*TOC1*) promoter. The rhythmic oscillation of H3 acetylation, as well as binding of chromatin remodeling factors, closely follows the circadian expression of the *TOC1* gene mediated by circadian clock associated 1 (CCA1) [148]. Collectively, these and other data strongly suggest a conserved interspecies molecular mechanism that couples light signal transduction and circadian rhythms with chromatin remodeling. Here we give a general overview of PTMs involved in the modulation of chromatin structure of circadian and light-dependent genes in the WCC model system, which may be considered as functional ancestor of the core clock system in mammals, plants and flies.

### 4.1. Chromatin Acetylation

Specific interactions between light-responsive regions (LRR) of the light-dependent genes and WCC have been demonstrated *in vivo* [27]. *In vitro* DNA binding assays using recombinant zinc-finger domains of WC-1, NIT-2, and CYS-4 did not preferentially bind to their specific GATA repeats on naked DNA segments consisting of target promoters [149]. This suggested the existence of other not yet described factors necessary in this interaction might be important in conferring specificity to the response. The correlation between chromatin remodeling and light responses clarified this point. The first evidence of this correlation was observed at histone H3 transient acetylation in the promoter region of *al-3* [130]. An increase in acetylation was observed 20 min after multiple light pulses delivered to wild-type *Neurospora* strains in different light conditions, with a transient kinetic comparable to that of *al-3* mRNA [150]. Lysine 14 (K14) has been shown to be involved in rhythmic acetylation after light pulse. Correlations between acetylation of acH3 K14 and transcriptional activation was confirmed in several other systems [151,152]. This was the first evidence that *Neurospora* light signal transduction involves chromatin modifications. Conversantly analysis of the acetylation state of core histones including AcH3 K9/K14 at the LRE region of the *frq* promoter did not show changes over the circadian cycle. Though this apparently contrasts with descriptions of the acetylation state of core histones at LRE regions, the discrepancy between these results may be due to differences in the loci examined and the regions therein[130,153].

**Figure 4:**
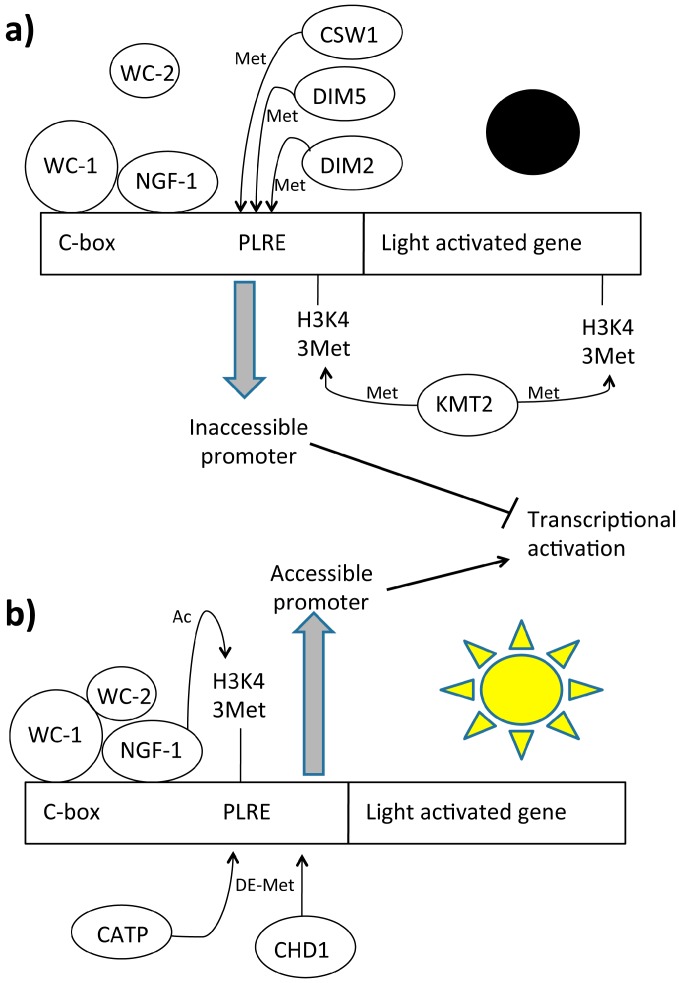
Light dependent chromatin remodeling on light activable genes. Legend: C-box: DNA box for protein specific interaction, CATP: Clock ATPase, CHD1: Chromo-domain helicase DNA-binding protein, CSW1: Clockswitch protein, DIM: Defective in protein methylation, KMT2: Histone methyltransferase Set1, NGF1: histone acetyltransferase homologous of GNC-5, PLRE: Proximal light-regulated element, WC-1: White Collar 1 protein, WC-2: White Collar 2 protein. (**a**) In dark conditions the methylases DIM2, DIM5, CSW1 and KMT2 generate a closed chromatin structure that prevents gene transcription; (**b**) Under light irradiation, NGF-1 acetylates H3K14 residues of light dependent regulatory elements of light activated genes. At the same time several demethylases such CHD1 and CATP remove the methyl groups added during the “dark conditions”, generating a more accessible chromatin structure.

A correlation between the transient acetylation of AcH3K14 and WCC activity was also demonstrated by the loss in light-dependent chromatin acetylation in a *wc-1 null* strain. Moreover, a *Neurospora h3^k14q^* mutant, who carried an ectopic mutant copy of histone H3 (*H3K14Q*), engineered to substitute a lysine to glutamine at residue 14, showed the same light-dependent aberrant phenotype of the *wc-1 null* mutant [130]. Light-induced carotenogenesis was strongly impaired in the *hH3K14Q* strain as compared with the isogenic wild type and this was coupled with a loss of light-inducible *al-3* mRNA oscillation. *Neurospora* Gcn Five-1 (NGF-1), a histone acetyl transferase homolog of yeast Gcn5p has also been found directly responsible for K14 H3 acetylation. This correlation was observed in the *ngf-1^RIP1^* mutant strain in which the absence of NGF-1 HAT activity was coupled with a loss of histone acetylation at K14 of histone H3. Finally, *ngf-1^RIP1^* and *wc-1 null* mutants exhibited identical phenotypes, suggesting that they are affected in the same signal transduction pathway. NGF-1 needs to physically interact with WC-1 in order to exert its own HAT activity [129]. Although this complex was preassembled in the dark, NGF-1 acetylated histones only after multiple light pulses (Figure 4). The more reasonable explanation is that, after multiple light pulses, a conformation shift in the region of the LOV domain of WC-1 changes the NGF-1 position on the nucleosome, thus activating its HAT function [154]. NGF-1 is unable to acetylate histones without correct binding with the C-term region of WC-1. It has been found that the LXXLL consensus sequence of WC-1 is involved in the interaction with NGF-1. This is a conserved consensus used for the binding between nuclear receptors and coactivators [155,156]. This means that WC-1 acts as a nuclear receptor. This was the first time that this mechanism was observed in fungi and the first demonstration of WCC-mediated chromatin remodeling in light-dependent events in *N. crassa* [157].

### 4.2. Chromatin Remodeling Enzymes

Multiple evidences suggest that dynamic histone accumulation and modification are under the control of the circadian cycle. Mouse Period 1 and Period 2 (*mPer1* and *mPer2*) genes, which encode central clock components functionally analogous to FRQ, have rhythmic AcH3 (K9, K14) modifications, and RNA polymerase II (Pol II) binds rhythmically to these promoters [144,151]. Since the mammalian clock gene *mPer2* has promoter methylation, chromatin remodeling and histone occupancy at the *frq* promoter region were analyzed. Transcriptional regulation at the *frq* promoter occurs through binding of the WC proteins to Clock box (C box) required for rhythmic oscillation in constant darkness. It occurs by binding with proximal light-regulated elements (PLREs) as well, necessary for *frq* light-dependent activation [24,51]. Occupancy of the *frq* promoter by WCC and DNase hypersensitivity of this region show circadian oscillation, contrasting with the common idea of a stable, preassembled and obligated WCC in all daily conditions as described above [153,158]. ChIP assays showed rhythmic association of WC-2 but not of WC-1 at the C-box, indicating a dynamic regulation that includes complex formation or disassembly. Moreover, at the PLRE region it was observed that WC-1 but not WC-2 is present in all circadian times. This means that accessibility to DNA by transcription factors is cyclically regulated during the day.

The ATP-dependent chromatin-remodeling enzyme CSW-1 is thought to be responsible for the closing catalytic activity [153]. Molecular and genotypic data showed that CSW-1 negatively regulates WCC activity by changing chromatin accessibility at the *frq* promoter. Indeed, strains lacking CSW-1 show a relatively open chromatin structure at the *frq* promoter. CSW-1 facilitates chromatin compaction and decrease of WC-2 levels at the *frq* promoter, thus promoting its repression. Translated FRQ cooperates to increase nucleosome occupancy and inhibit WCC binding at the C-box. Also, other enzymes that modify DNA methylation cooperate in chromatin compaction. The DNA methyltransferase (DNMT) DIM-2/HP1 (defective in methylation; heterochromatin protein-1) and DIM-5 are required for DNA methylation at *frq* promoter in circadian rhythmicity [159]. Deletion of *dim-2* results in a small phase defect (approximately 2 h) and changes in DNA methylation at *frq.* This indicates a link between DNA methylation and phasing of clock gene expression. Interestingly, DNA methylation at the *frq* promoter decreased with time in constant darkness, although not in an overtly circadian manner. Moreover, promoter methylation appeared greatly reduced in cultures grown in constant light for extended periods (48 h in light), providing evidence for another strong correlation between epigenetic modification and light signal transduction. Dim-5-dependent H3K9me3 is required for *frq* promoter methylation. Loss of DIM-5 can also suppress the hypermethylation phenotype of *chd1* and the mutant show a shorter phase advantage compared with dim-2 mutant period [159]. DIM-5 DNA activity is also established in response to the light and loss of H3K9me3 results in elevated WCC binding to the *frq* promoter and an increase in light activated *frq* expression. All together these data suggest that H3K9me3 appears to be involved light-mediated expression and is likely more important in the diurnal expression confirming that DNA methylation is required in the circadian entrainment. Antisense *qfr* RNA is also essential for normal DNA methylation in this region. This DNA methylation at *frq* is surprising. Indeed, in various *Neurospora* strains analyzed in many different conditions in which about 2% of cytosines are methylated, methylation exists exclusively as a relic of repeat-induced point (RIP) mutation and rDNA [160,161]. Since all these factors are indispensable for chromatin compacting, others are involved in chromatin opening for the next round of activation. Clock ATPase (CATP) counteracts CSW-1 activity, opening the chromatin structure and positively regulating nucleosome occupancy at the *frq* locus [162]. It has been demonstrated that CATP promotes the removal of nucleosomes at the *frq* locus and enhances WCC binding. CATP activity, but not CATP expression, shows circadian oscillation, counteracting the increase in nucleosome occupancy at the C-box. CATP specifically associates with the *frq* locus, suggesting that CATP regulates nucleosome occupancy directly on chromatin. The *catp^Ko^* strain shows a lower expression of FRQ, higher occupancy at the frq C-box, and minor WCC occupancy during the day as compared with the wild-typestrain. Though it is not clear how CATP works, both the ATPase domain and the bromodomain of CATP are essential for its function in the clock.

Chromo-domain helicase DNA-binding protein 1 (CHD1), another chromatin remodeler, has been found to be involved in *frq* chromatin remodeling through which it promotes the open state of chromatin [158]. Loss of CHD1 generates hypermethylation on the *frq* locus. It was demonstrated that CHD1 is required for maintaining regular high amplitude rhythms of WC-2 binding, which contributes to the apparent changes in chromatin structure. DNA methylation has been also found in the promoter of *wc-1* gene in the *dchd1* mutant strain but absent in the wild-type strain. These data suggest that CHD1 acts as a demethyltransferase that counteracts DIM2 and *qfr* activity, though the molecular mechanism is not clear. Finally, these data highlight that DNA and histone methylation modulates light responses and circadian rhythms. Figure 4 illustrates the chromatin rearrangements on light-activated genes. Table 3 presents the chromatin remodelers and their activity.

**Table 3:** Putative modifications in *frq* promoter (PLRE and C-BOX).

Enzyme	Modification	Target	Activation	Deactivation
NGF1	Acetylation	H3K9–H3K14	X	-
DIM-2	Methylation	Undefined	-	X
DIM-5	Methylation	H3K9	-	X
CSW-1	De-Acetylation	Undefined	-	X
KMT2	3Methylation	H3K4	-	X
CATP	Demethylation	Undefined	X	-
CHD1	Demethylation	Undefined	X	-

Every enzyme is tagged with a specific feature as “activation” or “deactivation” by marking with an X in the proper column.

### 4.3. Chromatin Methylation

Other factors cooperate with CSW-1 for chromatin compaction. The KMT2 enzyme, called SET1 (FGSC15827), has been isolated as a candidate for circadian and light-dependent histone methylation [163]. SET1 is part of a large holoenzymatic complex (complex associated with SET1 [COMPASS]) that contains several additional subunits involved in H3K4 methylation [164]. Strains lacking *set1* gene or a subset of other SET1/COMPASS subunits have an apparent arrhythmic clock phenotype on race tubes. Moreover, in analysis at transcriptional and translational levels confirmed the defect of *frq* in the *set1* mutant strain indicating that H3K4 methylation is required for efficient feedback inhibition of the central clock gene.

ChIP data indicate that H3K4me3 is present at the *frq* locus throughout the entire circadian cycle, with peaks occurring during the transcriptional repressive phase (CT9 to CT13) corresponding to the repressive phase of *frq* transcription. H3K4me3 is predominantly present in the *frq*-coding region, which is consistent with reports that H3K4 methylation is normally localized to coding regions and the transcriptional start site of genes [165]. This methylation is strongly regulated by SET1. Indeed, there is a delay in the temporal H3K4 methylation of the *frq* promoter in Δ*set1* mutant strains compared with wild-type strains. This delay increases the amplitude of *frq* expression under circadian and light-dependent conditions. This further suggests that H3K4me3 may play a role in the down-regulation of *frq.* Moreover, lack of functional SET1 causes a temporal alteration of rhythms in conidia formation.A defect in WC-2 binding to the C-box element in Δ*set1* mutant has been observed as consequence of the altered histone methylation state.

In wild-type *Neurospora* strains, no changes in H3K4 methylation were observed after exposure to light for 15 min at the PLRE. After 30 min, a time corresponding to the start of VIVID (VVD)-dependent light repression, the H3K4me3 levels rose and remained elevated, followed by a slow and steady decrease over the course of the next 2 h. This suggests that SET-1 can play a role also in light-dependent processes. These data confirm that histone methylation is necessary for modulating circadian and light responses by chromatin compaction. Figure 4 summarizes the factors involved in Chromatin methylation.

## 5. Conclusions

**Figure 5:**
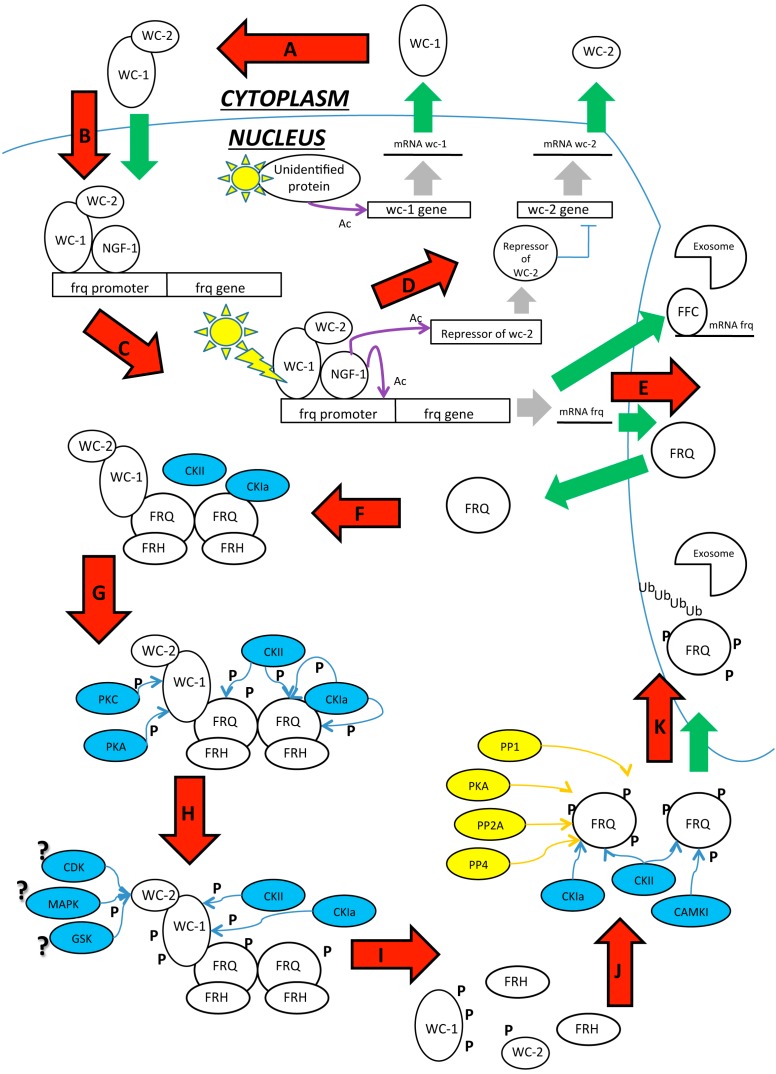
Scheme reporting the major PTM identified in the process of the circadian clock in *N. crassa*. This figure summarizes the single figures showed in the paper. Legend and Symbols: ? = Supposed, Ac: Acetyl group, Blue arrows: Phosphorylation, Blue circles: Kinases, Green arrows: import/export, Grey arrows: transcription, P: Phosphate group, Ub: Ubiquitin, Violet arrows: Acetylation, Yellow arrows: De-phosphorylation, Yellow circles: De-phosphorylates. (**A**) WC-1 and WC-2 presumably dimerize in the cytoplasm where they form the WCC that shuttlesinto the nucleus; (**B**) Here, the WCC recruits the inactive acetyltransferase NGF1 and binds the promoter of light-dependent genes; (**C**) After light pulse, the WCC changes conformation due to the LOV domain of WC-1, NGF1 is positioned onto the chromatin of the target genes *vvd* and *al-3*, and histone acetylation relaxes the chromatin. *Frq* mRNA is transcribed as well; (**D**) The WCC also activates the transcription of an unidentified repressor of the *wc-2* gene that blocks the production of WC-2 protein, generating a negative feedback on the WCC; (**E**) *Frq* mRNA is exported into the cytoplasm where it is translated. The hypophosphorylated FRQ is rapidly imported into the nucleus; (**F**) FRQ homodimerizes and recruits FRH that ensures proper FRQ folding. FRQ also recruits the kinases CKIa and CKII, generating the FFC. Finally, the FFC physically interacts with the WCC. High levels of FFC are responsible for *frq* mRNA degradation by targeting the transcript to the exosome (**E**); (**G**) CKIa and CKII phosphorylate the FRQ C-terminal domain. PKA and PKC phosphorylate WC-1 on S990 and S995, which is signal that targets the other serines for subsequent phosphorylation; (**H**) CKIa and CKII start to phosphorylate WC-1 at S992, S994, S998. WC-2 S443 is targeted by undefined FRQ-independent kinases; (**I**) Phosphate groups abolish the acidic properties of the FRQ N-terminal domain, disassembling the FFC. Phosphorylation also inhibits the interaction between FFC and WCC, causing the loop to close; (**J**) FRQ exposes its PEST domains and other phosphorylation sites. Numerous dephosphorylases contrast the kinase activity, stabilizing the protein and regulating FRQ half-life. The hyperphosphorylated FRQ is ubiquitinated and targeted for proteasomal degradation.

Post Translational Modifications (PTMs) generally influence oscillation of clock components and these modifications are strongly conserved among species [166,167]. In this review we discussed how PTMs can influence light responses and circadian rhythms in *N. crassa* and how they are involved in protein activity and chromatin remodeling. As shown in Figure 5 we propose a model on the basis of the data discussed above.

In constant darkness newly synthesized WC-1 and WC-2 dimerizes in the cytoplasm to form the WCC complex. Hyperphosphorylated FRQ seems to be necessary for assembling the WCC but its actual function remains to be elucidated. WCC translocates into the nucleus and binds the clock-controlled *frequency* promoter gene at the C-box. The hypophosphorylated WCC is better able to bind the promoter of clock-controlled genes, leading to their transcriptional activation. At the same time, the WCC activates a still uncharacterized WC-2 repressor that inhibits *wc-2* gene expression, generating a negative feedback loop in WCC production. After transcriptional activation, *frq* mRNA is exported into the cytoplasm and translated. The hypophosphorylated FRQ protein is then able to translocate into the nucleus and homodimerizes via its N-terminal domain. The homodimeric FRQ protein forms a complex with FRQ-interacting RNA helicase (FRH), termed the FRQ–FRH complex (FFC), which functions similarly to PERs and CRYs in mammals. This complex physically interacts with the WCC and it recruits several kinases, including CKa, CKb, CKII PKA and GSK3β, which balance FRQ circadian stability.

At the same time, PKA, the WCC priming kinase, phosphorylates WC-1 at S990 in an FRQ-independent pathway, driving the WC-1 phosphorylation mediated by CKa and CKII in a FRQ-dependent manner. FRQ changes its conformation after the first phosphorylation at the N-terminal domain, resulting in disassembly of the FCC complex. FRQ then exposes the PEST domain and other phosphorylating sites. WC-1 is subsequently phosphorylated at S992, S994, S998 sites close to the zinc-finger domain required for WC-1 dark activation. WC-2 is target of many not yet defined phosphorylation. Many phosphatases, including PP1, PP2a, and PP4, counteract kinase activities, stabilize the FRQ protein, and regulate the timing of its turnover. The hyperphosphorylated FRQ is exported into the cytoplasm, thereby driving the WCC shuttling and ensuring the FRQ-dependent clearance of the WC-1 from the nucleus. Finally, FRQ is ubiquitinated and targeted to the proteasome by F-box and WD40 repeat-containing protein 1 (FWD-1). Since phosphorylated WCC is no longer able to activate gene transcription, the FRQ level drastically decreases and the negative loop closes. WC-1 is recycled for a new circadian cycle in constant darkness.

Transcription of clock-controlled genes is also affected by DNA methylation, histone modifications, and nucleosome assembly/exchange. Occupancy of the *frq* promoter at the *C box* by the WCC and DNase hypersensitivity of this region shows circadian oscillation as well. The ATP-dependent chromatin remodeling enzyme Clockswitch (CSW-1), the DNA methyltransfrase DIM-2/HP1, the H3K9me3 dependent DIM-5, the antisense transcript *qrf*, the histone H3K4 methyltransferase SET1, and the translated FRQ all cooperate to compact the chromatin. They are counteracted by Clock ATPase (CATP) and CHD-1 that cooperate to open the chromatin structure, allowing the exposure of the C-box or light responsive elements (LREs). Moreover, the WCC also recruits the SWI/SNF (SWItch/Sucrose NonFermentable) complex that aids to remove the nucleosome from the *C box* and bends the DNA to bring the TSS close to the C box and the WCC, promoting transcriptional activation [168]. SWI/SNF is an ATP-dependent chromatin-remodeling complex that alters chromatin structure and helps in transcriptional activation, DNA repair, recombination and segregation [169,170].

In constant darkness conditions, the WCC also recruits histone acetyltransferase NGF-1 and binds dependent gene promoters at the LREs *al-3* or *vivid*. Probably, NGF-1 is recruited also onto the *frq* LRE, but this interaction has not yet been demonstrated. After multiple light pulses, the WCC undergoes LOV-mediated conformation and positions NGF-1 onto the promoter of the target genes. The light-dependent H3K14 acetylation mediated by NGF-1 activates light-dependent gene transcription. The H3K14 acetylation decreases in amplitude when WCC becomes hyperphosphorylated in a light-dependent manner. The phosphorylation is mediated by PKC and FRQ-complex and dissociates the WCC from the promoter of its target genes and reduces promoter acetylation, causing the transcription to switch off. This leads to a decrease in *frq* mRNA and protein amount, which closes the light-dependent negative loop. After the light/dark switch, reactivation of WC-1 can generate a new circadian cycle or light-dependent responses.

The conservation of the posttranslational mechanisms is remarkable among different eukaryotic circadian systems from prokaryotes to humans [82].

Cyanobacteria are the most ancient example of Post Translational Modifications-mediated circadian oscillation in which the cell-self sustained circadian period length and its stability are derived from the ATPase activity of KaiC kinase [171,172,173].

There are several strands of evidence supporting the idea of a conserved molecular mechanism between mammals and *Neurospora*. Similar to FRQ/WCs, animal and plant clock proteins are regulated at post-translational level. Period proteins, members of the negative loop in the fly (PER) and mammalian (mPER2) circadian clock, are progressively phosphorylated and degraded [174,175]. The mammalian Bmal1, Clock and Cry also exhibit robust rhythms in their phosphorylation profiles [176,177,178,179,180,181].

In addition, FRQ and the animal PER proteins are phosphorylated by CKI and CKII, dephosphorylated by the same phosphatases, and degraded by the ubiquitin/proteasome system using a conserved E3 ubiquitin ligase [75,76,77,78,79,90,91,92,93,94,95,96,97,98,99,100,101,102,103,104,105,106,107,108,109,110,111,112,113,114].

Furthermore, a antisense of the *per* RNA has recently been discovered that should work as a *qrf* [182]. All together these evidences further confirmed the presence of a conserved mechanism involved in circadian clock and light signal transduction across living kingdoms. This holds interest for research in *Neurospora*, which, because of its rapid life cycle, availability of a large number of mutations and ease of manipulation, provides a useful model system for studying circadian clock and light signal transduction pathways. This makes *N. crassa* a powerful tool for improving our understanding of the epigenetic and posttranslational regulations of circadian rhythms that are highly conserved across species.
